# CD8^+^ T Cells from SIV Elite Controller Macaques Recognize Mamu-B*08-Bound Epitopes and Select for Widespread Viral Variation

**DOI:** 10.1371/journal.pone.0001152

**Published:** 2007-11-14

**Authors:** John T. Loffredo, Thomas C. Friedrich, Enrique J. León, Jason J. Stephany, Denise S. Rodrigues, Sean P. Spencer, Alex T. Bean, Dominic R. Beal, Benjamin J. Burwitz, Richard A. Rudersdorf, Lyle T. Wallace, Shari M. Piaskowski, Gemma E. May, John Sidney, Emma Gostick, Nancy A. Wilson, David A. Price, Esper G. Kallas, Helen Piontkivska, Austin L. Hughes, Alessandro Sette, David I. Watkins

**Affiliations:** 1 Wisconsin National Primate Research Center (WNPRC), University of Wisconsin-Madison, Madison, Wisconsin, United States of America; 2 Division of Infectious Diseases, Federal University of São Paulo, São Paulo, Brazil; 3 Department of Pathology and Laboratory Medicine, University of Wisconsin-Madison, Madison, Wisconsin, United States of America; 4 Division of Vaccine Discovery, La Jolla Institute for Allergy and Immunology, La Jolla, California, United States of America; 5 Weatherall Institute of Molecular Medicine, University of Oxford, John Radcliffe Hospital, Oxford, United Kindgom; 6 Department of Biological Sciences, Kent State University, Kent, Ohio, United States of America; 7 Department of Biological Sciences, University of South Carolina, Columbia, South Carolina, United States of America; University of California at San Francisco, United States of America

## Abstract

**Background:**

It is generally accepted that CD8^+^ T cell responses play an important role in control of immunodeficiency virus replication. The association of *HLA-B27* and -*B57* with control of viremia supports this conclusion. However, specific correlates of viral control in individuals expressing these alleles have been difficult to define. We recently reported that transient *in vivo* CD8^+^ cell depletion in simian immunodeficiency virus (SIV)-infected elite controller (EC) macaques resulted in a brief period of viral recrudescence. SIV replication was rapidly controlled with the reappearance of CD8^+^ cells, implicating that these cells actively suppress viral replication in ECs.

**Methods and Findings:**

Here we show that three ECs in that study made at least seven robust CD8^+^ T cell responses directed against novel epitopes in Vif, Rev, and Nef restricted by the MHC class I molecule Mamu-B*08. Two of these Mamu-B*08-positive animals subsequently lost control of SIV replication. Their breakthrough virus harbored substitutions in multiple Mamu-B*08-restricted epitopes. Indeed, we found evidence for selection pressure mediated by Mamu-B*08-restricted CD8^+^ T cells in all of the newly identified epitopes in a cohort of chronically infected macaques.

**Conclusions:**

Together, our data suggest that Mamu-B*08-restricted CD8^+^ T cell responses effectively control replication of pathogenic SIV_mac_239. All seven regions encoding Mamu-B*08-restricted CD8^+^ T cell epitopes also exhibit amino acid replacements typically seen only in the presence of Mamu-B*08, suggesting that the variation we observe is indeed selected by CD8^+^ T cell responses. SIV_mac_239 infection of Indian rhesus macaques expressing Mamu-B*08 may therefore provide an animal model for understanding CD8^+^ T cell-mediated control of HIV replication in humans.

## Introduction

Several lines of evidence suggest that CD8^+^ T cells play a key role in immune control of immunodeficiency virus replication. The reduction in acute viremia is associated with the appearance of CD8^+^ T cell responses in both HIV-infected humans [1], [2] and SIV-infected macaques [3], [4], though recent experiments suggest that this reduction could also be due to the acute depletion of memory CD4^+^ T cells that are the preferred targets for infection [5], [6]. Expression of particular HLA/MHC class I alleles is associated with reduced plasma viremia and/or slower disease progression in humans [7]–[12] and macaques [13]–[17]. CD8^+^ T cell responses also exert selective pressure on replicating viruses, resulting in the emergence of variants that escape immune detection in both HIV and SIV infection [18]–[27]. Most strikingly, transient depletion of circulating CD8^+^ lymphocytes in SIV-infected macaques results in dramatic increases in plasma viremia [28]–[30]. These findings suggest that inducing CD8^+^ T cell responses will be an important component of AIDS vaccine strategies. However, because most HIV- and SIV-infected individuals mount CD8^+^ T cell responses, and the majority of these individuals progress to AIDS, it is clear that the presence of strong responses alone is not sufficient to control viral replication or delay disease progression. Therefore, it is important to define attributes that might distinguish effective CD8^+^ T cell responses from ineffective ones.

In the effort to define such correlates of control for CD8^+^ T cells, there has been considerable interest in “elite controllers” (ECs), rare individuals who spontaneously control HIV viremia to levels below the detection threshold of current assays (<50 vRNA copy Eq/ml plasma) [31]. Effective control of HIV replication in some such individuals could be mediated by determinants other than cellular immunity, such as infection with attenuated viruses [32]–[35], polymorphisms in host genes outside the MHC [36]–[40], or autoimmune antibodies directed against the CCR5 coreceptor [41], [42]. However, there is strong evidence suggesting that particular CD8^+^ T cell responses play a major role in effective viremia control in at least some ECs. Vigorous CD8^+^ T cell responses have been observed in individuals with non-progressive infection [43], [44]. Investigation of the phenotypes of HIV-specific CD8^+^ T cells has suggested that controllers retain effector functions that are lost in progressors [45]–[48]. The MHC class I alleles *HLA-B27* and HLA-B57/B*5801 are over-represented in cohorts of ECs, suggesting that the cellular immune responses they restrict contribute to immune containment of viral replication [7]–[12]. In support of this suggestion, viral escape from the immunodominant response to the HLA-B27-restricted epitope Gag_263–272_KK10 has been associated with loss of control of viral replication [21], [49]–[51]. The role of viral evolution and particular epitope-specific responses in control associated with *HLA-B57*, however, remains less clear [52].

Studies of elite HIV control in humans are limited by drawbacks inherent to research in populations infected with diverse virus strains. An animal model of effective viremia control would complement these studies by offering an example of successful immune containment of pathogenic AIDS virus replication while allowing direct control over key variables such as virus strain, host genotype, and timing and route of infection. To this end, several studies have noted relationships between MHC class I genotypes and effective control of viremia in cohorts of SIV-infected rhesus macaques that mirror those seen in human EC cohorts. Expression of the common MHC class I allele Mamu-A*01 has been observed to lower set-point viremia in vaccinated and non-vaccinated macaques in several studies [13]–[15], [53]. More strikingly, we have recently reported an association between a different high-frequency MHC class I allele, Mamu-B*17, and an even greater reduction of chronic phase viremia [17]. Mamu-B*17, but not Mamu-A*01, was also over-represented in a cohort of EC macaques that maintained chronic phase SIV viremia <1,000 vRNA copy Eq/ml [17], [54].

To test the hypothesis that CD8^+^ T cell responses are involved in the ongoing control of viremia in EC macaques, we recently treated six ECs (four Mamu-B*17-positive, two *Mamu*-B*17-negative) with the monoclonal antibody cM-T807, which transiently depletes circulating CD8^+^ lymphocytes [55]. This treatment resulted in a brief viral recrudescence in all six ECs, after which viremia declined back to near pre-depletion levels. Analysis of the returning wave of CD8^+^ T cells showed different degrees of expansion in epitope-specific populations in each animal. We noted that the two Mamu-B*17-negative macaques shared expanding CD8^+^ T cell populations that recognized previously unmapped epitopes in the SIV proteins Vif and Nef. Here we report minimal optimal sequences for these novel epitopes and show that they are presented by the MHC class I molecule Mamu-B*08.

We recently discovered that Mamu-B*08 is strongly associated with control of SIV_mac_239 replication [54]. We have extended this finding, showing that Mamu-B*08 presents at least seven CD8^+^ T cell epitopes derived from SIV_mac_239. Furthermore, we show that the sequences of viruses replicating during chronic infection have substitutions in several Mamu-B*08-restricted epitopes, indicating that CD8^+^ T cells restricted by this molecule exert selective pressure on the virus at multiple sites. We also report the construction of Mamu-B*08 tetramers loaded with each of the newly discovered epitope peptides, enabling us to determine the chronic-phase immunodominance hierarchy of these responses.

## Methods

### Animals and viruses

Indian rhesus macaques (*Macaca mulatta*) were initially identified as Mamu-B*08-positive by analysis of MHC class I cDNA libraries. PCR with sequence-specific primers (PCR-SSP) was used as previously described [54] for confirmation and to identify additional Mamu-B*08-positive macaques. Animals were screened for the presence of nine additional MHC class I alleles (Mamu-A*01, -A*02, -A*08, -A*11, -B*01, -B*03, -B*04, -B*17, and -B*29) using PCR-SSP as previously described [56], [57].

Animals were infected with the pathogenic molecular clone SIV_mac_239 [58] (GenBank accession M33262), with the exception of macaque r99006, which was infected with an SIV_mac_239 recombinant bearing escape mutations in three CD8^+^ T cell epitopes [59], [60]. Animals' plasma virus concentrations were monitored by quantitative PCR as previously described [61], [62].

EC macaques r00078, r01064, and r98016 were transiently depleted of CD8^+^ lymphocytes as part of a prior study [55]. These three ECs and animal r99006 were subsequently rechallenged intravenously with 100 TCID_50_ SIV_mac_239 with no effect on viremia or the frequency of SIV-specific CD8^+^ T cells (Friedrich *et al*., unpublished data).

SIV-infected animals were maintained at the National Primate Research Center (University of Wisconsin-Madison, Madison, WI) and cared for according to the regulations and guidelines of the University of Wisconsin Institutional Animal Care and Use Committee.

### Construction of MHC class I cDNA libraries

Total RNA from animals r00078, r01064, and r98016 was isolated from ∼3×10^7^ cells from B-lymphoblastoid cell lines (BLCL) using the RNeasy Protect Mini Kit (QIAGEN, Valencia, CA). For each animal, ∼3 µg mRNA was isolated from 150 µg total RNA using the Oligotex Midi Kit (QIAGEN). One microgram of mRNA from each animal served as the template for first strand cDNA synthesis, using the SuperScript plasmid system for cDNA synthesis and cloning (Invitrogen, Carlsbad, CA) by following the manufacturer's instructions. Size-fractionated cDNA containing SalI and NotI restriction endonuclease cohesive ends was ligated into the multiple cloning site of pCMV.SPORT6 and used to transform DH5α chemically competent *E. coli* (Invitrogen). Recombinant plasmids containing cDNA were isolated from ∼5×10^5 ^ampicillin resistant colonies and purified using the HiSpeed Plasmid Midi kit (QIAGEN). Five micrograms of plasmid DNA from each macaque's library served as the target DNA for hybridization to a biotinylated oligonucleotide, 5′-CGGAGATCAYRCTGACVTGGC-3′. The sequence of the capture oligonucleotide was derived from a highly conserved region of the MHC class I alpha-3 domain. The GeneTrapper cDNA positive selection system (Invitrogen) was then used to enrich the cDNA library for MHC class I alleles.

More than 150 MHC class I clones were captured and sequenced from each library. Sequencing was performed on an ABI 3730 DNA Analyzer (Applied Biosystems, Foster City, CA). Full-length sequences of MHC class I cDNAs were obtained by using four forward and four reverse primers. The forward primers were: SP6 (5′-GGCCTATTTAGGTGACACTATAG-3′), C/1+ (5′-GCAGATACCTGGAGAACGGG-3′), IV (5′-GGAACCTTCCAGAAGTGGG-3′), and 3′UTR (5′-CAGGGCTCTGATGTGTCTCTCACG-3′). The reverse primers were: T7 (5′-TAATACGACTCACTATAGGG-3′), E2 (5′-CYCCACCTCCTCACATKATGC-3′), F1 (5′-CCAGGTCAGTGTGATCTCCG-3′), and G1 (5′-ATGTAATCCTTGCCGTCGTA-3′). Sequences were analyzed using CodonCode Aligner version 1.6.3 (CodonCode, Deadham, MA). MHC class I alleles were considered part of the cDNA library after at least two copies were verified by sequencing. Novel MHC I sequences were given GenBank accession numbers: Mamu-A6*0103 (EF602318), referred to as Mamu-A*15 in Table 1, Mamu-B*31 (EF602319), and Mamu-B*5102 (EF362450).

**Table 1:** Major histocompatibility complex class I profiles of three CD8^+^ cell-depleted EC macaques.

MHC class I	MHC class I animal profile^a^		
	r00078	r01064	r98016
***Mamu-A*** **alleles**		A*02	A*02
	A*0505		
		A*0507	A*0507
		A*07	A*07
	A*08		
		A*1302	A*1302
	A*1303		
		A*15 b	
***Mamu-B*** **alleles**	B*06 c	B*06 c	B*06 c
	B*08	B*08	B*08
		B*12	
			B*17
		B*22	
	B*29012		B*29012
	B*30	B*30	
		B*31	
		B*5102	
	B*53	B*53	
			B*6002
	B*64		

a) Profile constructed from cDNA libraries of at least 150 sequenced clones.

b) Updated nomenclature in GenBank = A6*0103 (EF602318).

c) Mamu-B*06 has not been detected by 1-D IEF, suggesting that it may not encode an expressed MHC class I protein [74].

### Generation and maintenance of SIV-specific CD8^+^ T cell lines

Peptide-specific CD8^+^ T cell lines were generated using previously described methods [62], [63]. Briefly, freshly isolated PBMC or CD8^+^ cell-enriched PBMC were used to start CD8^+^ T cell lines. Autologous B-lymphoblastoid cell lines (BLCL) were used as antigen presenting cells (APCs). BLCL were pulsed with 1 µM relevant SIV-specific peptide for 1 to 2 hours at 37°C, washed twice, and irradiated (9,000 rads). BLCL were then mixed with either whole or CD8^+^ cell-enriched PBMC at a ratio of 1∶1 in RPMI 1640 (Cambrex, Walkersville, MD) supplemented with L-glutamine (Mediatech, Herndon, VA), antibiotic-antimycotic solution (Mediatech), and 15% fetal bovine serum (FBS; HyClone, Logan, UT) (R15) with 10 ng/ml of recombinant human interleukin-7 (Sigma-Aldrich, St. Louis, MO) and incubated for 48 hours. Cells were cultured with R15 containing 100 Units of interleukin-2/ml (NIH AIDS Research and Reference Reagent Program, Germantown, MD) (R15-100) every 3 to 5 days thereafter. The CD8^+^ T cell lines were restimulated using peptide-pulsed, irradiated BLCL every 7 to 14 days. CD8^+^ T cell lines were tested for epitope specificity after >14 days in culture by either intracellular cytokine staining (ICS) or MHC class I tetramer assays as previously described [62]-[64]. MHC class I tetramers were constructed with minor modifications as previously described [64], [65].

### MHC class I transfectants

Transient expression of cloned MHC class I cDNA was achieved by electroporation of plasmid DNA into the MHC class I deficient human B-cell line 721.221 [66]. Briefly, 5 µg of plasmid DNA was added to 5×10^6^ 721.221 cells in 100 µl of Nucleofector™ Solution C and electroporated using program G-16 on a Nucleofector I device (Amaxa, Köln, Germany). Maximum cell surface expression occurred four days post-electroporation. The stable Mamu-B*08 transfectant was created as previously described [66], [67], except for the use of the Nucleofector I device (Amaxa) according to manufacturer's protocols.

MHC class I surface expression on stable and transient MHC class I transfectants was measured by W6/32 antibody surface staining. Staining was also performed on the 721.221 cells as a negative control and immortalized macaque B-cell lines (positive control). Approximately 0.5–1×10^5^ lymphocyte-gated events were acquired on a FACSCalibur (BD Biosciences, San Jose, CA) and analyzed using FlowJo version 8.4.5 (TreeStar, Ashland, OR).

### Intracellular cytokine staining (ICS) assay

TNF-α and IFN-γ intracellular cytokine staining (ICS) assays were performed on both freshly isolated PBMC and SIV-specific CD8^+^ T cell lines as previously described [55], [62], [63]. Briefly, each PBMC ICS test contained ∼5×10^5^ cells, while each CD8^+^ T cell line ICS test contained 2×10^5^ CD8^+^ T cells along with 1×10^5^ autologous BLCL. Individual peptides were used at a concentration of 5 µM or in serial ten-fold dilutions ranging from 5 µM to 5 pM. SIV peptide pools each contained ten 15-mer peptides overlapping by eleven amino acids. Approximately 0.5–1×10^5^ lymphocyte-gated events were acquired on a FACSCalibur (BD Biosciences) and analyzed using FlowJo 8.4.5 (TreeStar). All values were normalized by subtracting the background (cytokine-positive events in negative control samples of PBMC or CD8^+^ T cell lines in media without stimulation).

### IFN-γ Enzyme-Linked Immunospot (ELISPOT) assay

ELISPOT assays were performed as previously described [62]. Briefly, freshly isolated PBMC were used directly in precoated ELISpot^PLUS^ kits (MABTECH Inc, Mariemont, OH) for the detection of monkey IFN-γ according to manufacturer's protocols. 1×10^5^ PBMC were used per well and incubated 14–18 hours at 37°C in 5% CO_2_. Peptides were used at 10 µM or in serial ten-fold dilutions ranging from 10 µM to 10 pM. SIV peptide pools each contained ten 15-mer peptides overlapping by eleven amino acids. All tests were performed in either duplicate or triplicate.

Wells were imaged with an AID ELISPOT reader (AID, Strassberg, Germany), counted by AID EliSpot Reader version 3.2.3, and analyzed as previously described [62], [64]. Background (mean number of spot-forming cells [SFCs] in wells without peptide stimulation) was subtracted from each well on the plate. A response was considered positive if the mean number of SFCs from triplicate or duplicate sample wells exceeded background plus two standard deviations (SD). Assay results are shown as SFCs per 1×10^6^ PBMC. Responses <50 SFCs per 1×10^6^ PBMC were not considered positive.

### Sequencing of plasma viral RNA

Viral sequencing was performed essentially as previously described [55], [68]. Briefly, viral RNA was extracted from plasma using the QIAGEN MinElute kit. We used the QIAGEN One Step RT-PCR kit to amplify overlapping regions ∼300–800 nucleotides in length that spanned the SIV_mac_239 open reading frames (ORFs) *vif*, *rev*, or *nef*. The RT-PCR conditions for all amplicons were as follows: 50°C for 30 min; 95°C for 15 min; 45 cycles of 94°C for 30 s, 53°C for 1 min and 72°C for 150 s; and 68°C for 20 min. Cycling ramp rates were 2°C per second. The amplified cDNA was purified using the QIAGEN PCR purification kit. For some extremely low copy number samples (<100 vRNA copy Eq/ml) PCR products were directly cloned into the pCR®4-TOPO® vector using the TOPO-TA Cloning Kit (Invitrogen). Plasmids containing cloned sequences were purified using the QIAprep Spin Miniprep Kit (QIAGEN). Both strands of each amplicon were sequenced on a 3730 DNA Analyzer (Applied Biosystems). Sequences were assembled using Aligner version 1.6.3 (CodonCode). DNA sequences were conceptually translated and aligned to wild type SIV_mac_239 in MacVector 9.0 trial version (Accelrys, Burlington, MA).

### Statistical analysis of viral variation

The number of synonymous substitutions per synonymous site (*d_S_*) and the number of nonsynonymous substitutions per nonsynonymous site (*d_N_*) between the viral sequence obtained from each animal's circulating virus and the inoculum sequence (SIV_mac_239) were estimated using the method of Nei and Gojobori [69], and the results were analyzed as previously described [68], [70]. In the case of ambiguous nucleotides, we assumed equal occurrences of the possible nucleotides in the viral population in any given monkey. Note that, since the virus evolved independently in each monkey, comparisons of viral sequences with the inoculum are both statistically and phylogenetically independent [71].

## Results

### Mamu-B*17-negative ECs share robust responses to novel epitopes derived from Vif and Nef

While the majority of SIV ECs express Mamu-B*17 [17], several do not. In an attempt to define the immune correlates of control, we depleted CD8^+^ cells *in vivo* from four Mamu-B*17-positive and two Mamu-B*17-negative ECs [55]. In this transient CD8^+^ cell depletion experiment, particular virus-specific CD8^+^ T cells repopulating the periphery expanded above baseline frequencies. The set of epitopes recognized by expanding populations was different in each animal, but in each case previously subdominant populations showed a much greater relative expansion than dominant ones. We hypothesized that these expanding CD8^+^ T cell populations played an important role in the re-assertion of control over SIV replication.

**Figure 1:**
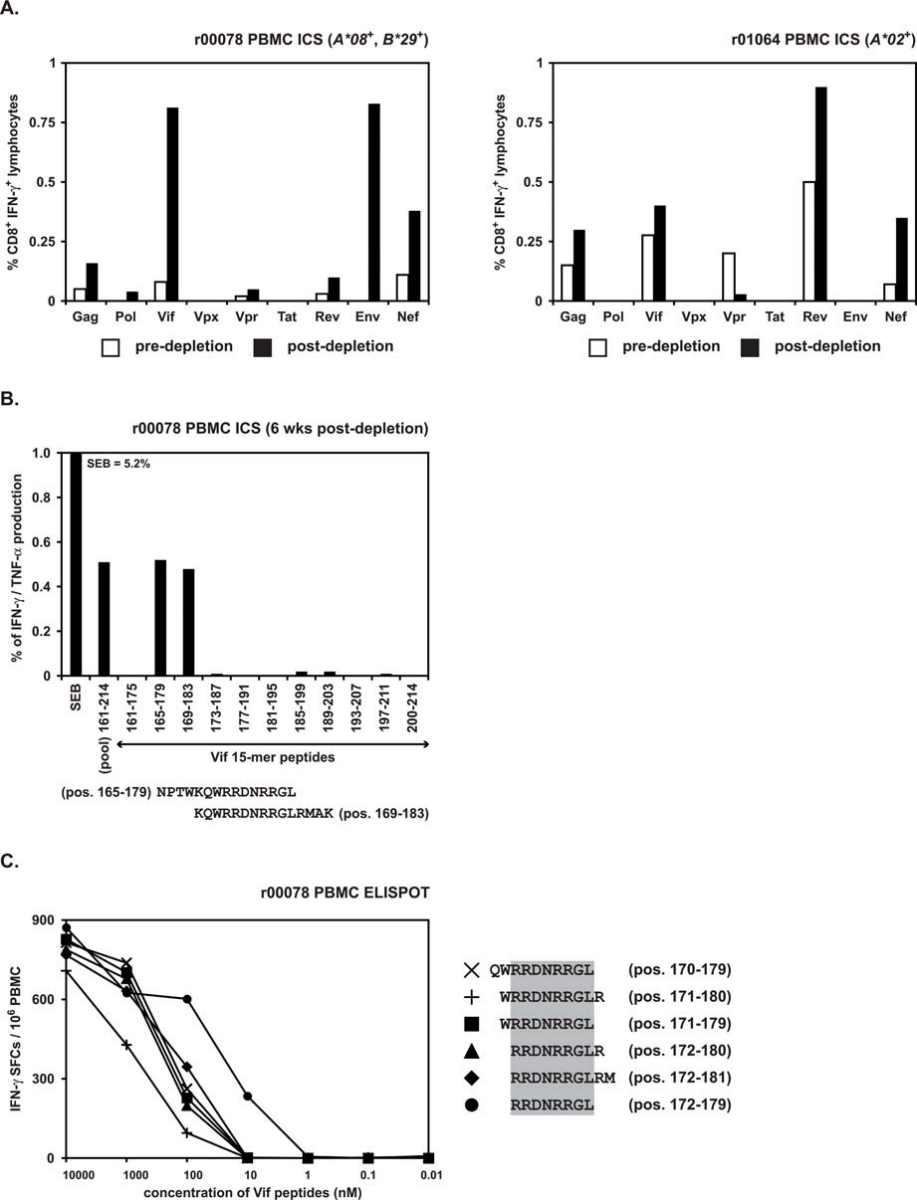
Identification and mapping of a novel CD8^+^ response directed against Vif that expanded in two CD8^+^ cell-depleted EC macaques, r00078 and r01064. MHC class I alleles detected by PCR-SSP are listed for each animal. A) Unknown CD8^+^ responses from *ex vivo* ICS assays using SIV peptide pools performed one month prior to (white bars) and four weeks after (black bars) CD8^+^ cell depletion. Responses to 15-mer pools that did not contain previously identified minimal optimal epitope sequences [64], [73], [82] are summed for each protein. Of particular interest was an unknown SIV-specific CD8^+^ response directed against Vif, which expanded in two Mamu-B*17-negative ECs r00078 and r01064. B) *Ex vivo* ICS deconvolution of peptide pool Vif E (positions 161–214) using r00078 PBMC demonstrated that the novel CD8^+^ epitope was located within two overlapping 15-mers in Vif (positions 165–183). C) *Ex vivo* IFN-γ ELISPOT using peptide dilutions in r00078 fine-mapped the novel CD8^+^ epitope to an 8-mer at positions 172–179 in Vif (RRDNRRGL). Mean values from triplicate wells were calculated for each peptide test.

Expanding CD8^+^ T cell populations in the four Mamu-B*17-positive ECs after depletion targeted epitopes in Vif and Nef [55]. Interestingly, expanding populations in the two Mamu-B*17-negative ECs, r00078 and r01064, also responded to peptides derived from these proteins (Fig. 1A). Indeed, both Mamu-B*17-negative ECs had large populations of CD8^+^ lymphocytes that recognized the same pool of ten Vif 15-mer peptides, Vif E (SIV_mac_239 Vif residues 161-214). To determine whether the animals might be recognizing the same epitope, we mapped the minimal optimal epitope recognized by both animals. First, we deconvoluted the 15-mer peptide pool, testing the ability of each individual 15-mer to stimulate IFN-γ secretion by the animals' PBMC in ICS assays. These experiments showed that the response to the pool was recapitulated by stimulation with either of two individual 15-mers (Fig. 1B). Since the responses to each 15-mer were very similar in magnitude for both animals, we reasoned that the minimal optimal epitope was contained within the region of overlap for the two peptides. IFN-γ ELISPOT assays using dilutions of candidate minimal optimal peptides showed the epitope recognized by each animal to be an 8-mer, RRDNRRGL, Vif_172–179_RL8 (Fig. 1C).

**Figure 2:**
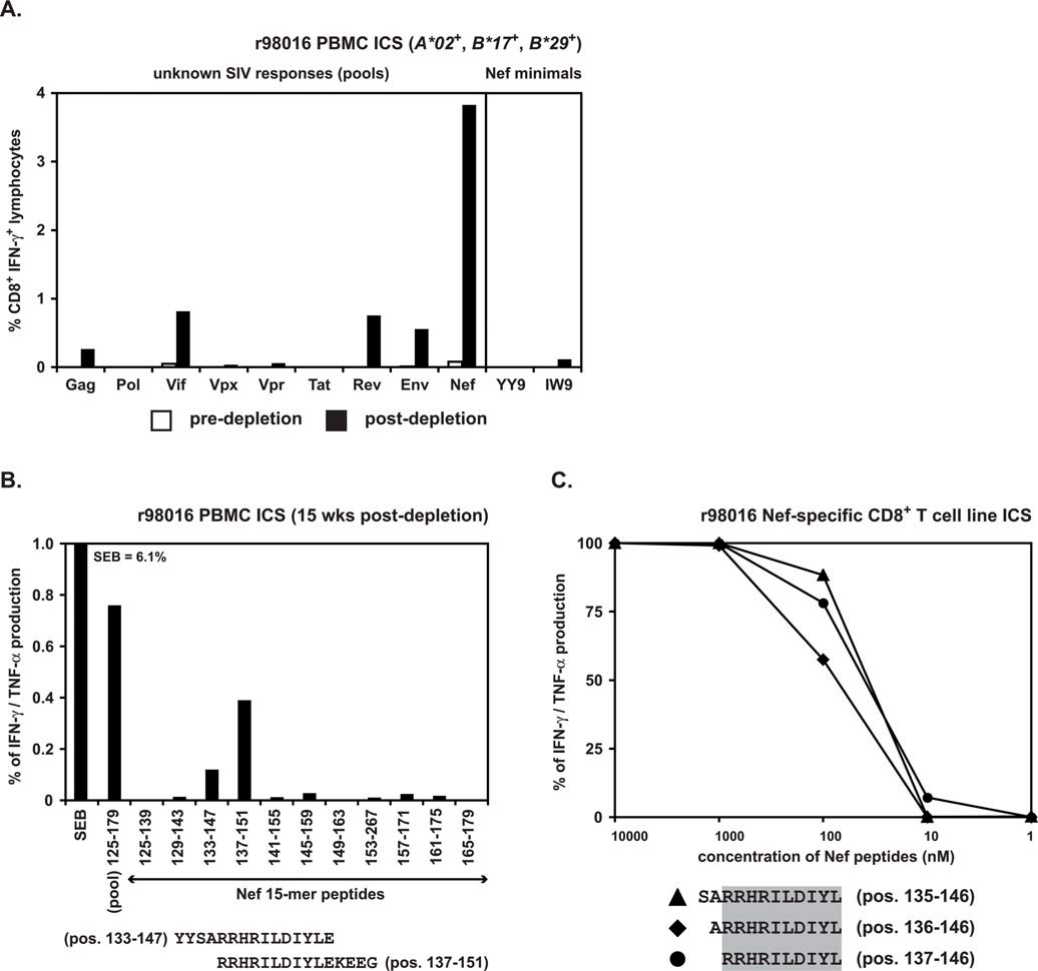
Identification and mapping of a novel CD8^+^ response directed against Nef that expanded in the CD8^+^ cell-depleted EC macaque r98016. MHC class I alleles detected by PCR-SSP are listed for r98016. A) Unknown CD8^+^ responses from *ex vivo* ICS assays using SIV peptide pools performed one month prior to (white bars) and four weeks after (black bars) CD8^+^ cell depletion. Responses to 15-mer pools that did not contain previously identified minimal optimal epitope sequences [i.e., Nef_159–167_YY9 and Nef_165–173_IW9] [64], [73], [82] are summed for each protein. Of particular interest was an unknown SIV-specific CD8^+^ response directed against Nef, which expanded in Mamu-B*17-positive EC r98016. B) *Ex vivo* ICS deconvolution of peptide pool Nef D (positions 125–179) using r98016 PBMC revealed that the novel CD8^+^ epitope was located within two overlapping 15-mers in Nef (positions 133–151). C) ICS results using peptide dilutions with a Nef-specific CD8^+^ T cell line from r98016 fine-mapped the novel CD8^+^ epitope to a 10-mer at positions 137–146 in Nef (RRHRILDIYL).

We also noted a strong response to an unidentified epitope in a third EC, r98016. More than 3% of this animal's returning CD8^+^ lymphocytes responded to a pool of Nef-derived 15-mers, Nef D (SIV_mac_239 Nef residues 125–179) (Fig. 2A). This peptide pool contains previously described dominant epitopes bound by Mamu-A*02 (Nef_159–167_YY9) [63], [72] and Mamu-B*17 (Nef_165–173_IW9) [16], [67], [73]. Surprisingly, although r98016 expressed both Mamu-A*02 and B*17, only ∼0.1% of its CD8^+^ lymphocytes recognized the Nef_165–173_IW9 peptide, while responses to Nef_159–167_YY9 were undetectable (Fig. 2A). The Mamu-A*02-negative, Mamu-B*17-negative EC r00078 also made a strong response to the same Nef pool after CD8^+^ cell depletion (0.26% CD8^+^ IFN-γ^+^, Fig. 1A and data not shown). We therefore hypothesized that these animals made a CD8^+^ T cell response to a novel epitope in Nef, which was not presented by either Mamu-A*02 or -B*17. To test this hypothesis, we first deconvoluted the response to Nef pool D in r98016 using ICS for IFN-γ and TNF-α. The strongest response to an individual peptide was stimulated by a single 15-mer, Nef_137–151_ (Fig. 2B). No 8-mer or 9-mer Nef peptide in this region elicited a similarly strong response (data not shown). To define the minimal optimal epitope we then tested the ability of serial ten-fold dilutions of larger candidate peptides (ten to twelve amino acids in length) to stimulate IFN-γ and TNF-α secretion in ICS assays using Nef-specific CD8^+^ T cell lines from animal r98016 (Fig. 2C). Although the 10-mer, 11-mer, and 12-mer all showed similar functional avidity, we defined the 10-mer as the minimal optimal, since it was the shortest peptide to stimulate the maximal response. The sequence of this peptide was RRHRILDIYL, Nef_137–146_RL10. IFN-γ ELISPOT assays using PBMC gave similar results, further suggesting that Nef_137–146_RL10 was the minimal optimal epitope recognized by r98016 (data not shown). PBMC from animals r00078 and r01064 also recognized overlapping 15-mer peptides containing this 10-mer sequence (see below), suggesting that all three ECs responded to this same epitope. MHC class I tetramer refolding subsequently confirmed that Nef_137–146_RL10 is the minimal optimal epitope bound by Mamu-B*08 (data not shown).

### CD8^+^ T cells from SIV EC macaques recognized novel Mamu-B*08-restricted epitopes

It appeared that all three ECs recognized the same novel Nef_137–146_RL10 epitope, RRHRILDIYL, while at least two animals, r00078 and r01064, recognized the novel Vif_172–179_RL8 epitope RRDNRRGL. Both these peptides had a diarginine motif at their N-termini and a leucine residue at their C-termini. Furthermore, BLCL derived from all three animals could present peptides to Vif_172–179_RL8-specific CD8^+^ T cell lines from animals r00078 and r01064 and also to Nef_137–146_RL10-specific CD8^+^ T cell lines generated from r98016 (data not shown). We therefore hypothesized that the novel epitopes were bound by the same, as yet unidentified, MHC class I molecule.

All ECs in the CD8^+^ cell depletion study had been screened for the presence of nine MHC class I alleles by sequence-specific PCR (PCR-SSP). Animals r00078, r01064, and r98016 did not share an allele detected in this screen (Figs. 1 and 2). PCR-SSP is useful as a high-throughput screen for the presence or absence of particular alleles of interest, but does not give a complete MHC class I genotype. In order to determine the full complement of MHC class I genes in each of the three ECs, we prepared cDNA libraries of MHC class I sequences from each animal. We sequenced at least 150 individually cloned cDNAs from these libraries for each EC. This number of clones is typically sufficient to identify the majority, if not all, of the MHC class I alleles expressed in an animal. Screening of the MHC class I cDNA libraries revealed two MHC class I alleles shared by all three ECs: Mamu-B*06 and Mamu-B*08 (Table 1). We had previously determined that Mamu-B*08 encodes a protein detectable by one-dimensional isoelectric focusing (1-D IEF), while Mamu-B*06 does not [74]. It was therefore likely that the restricting element for the two novel epitopes was encoded by Mamu-B*08.

**Figure 3:**
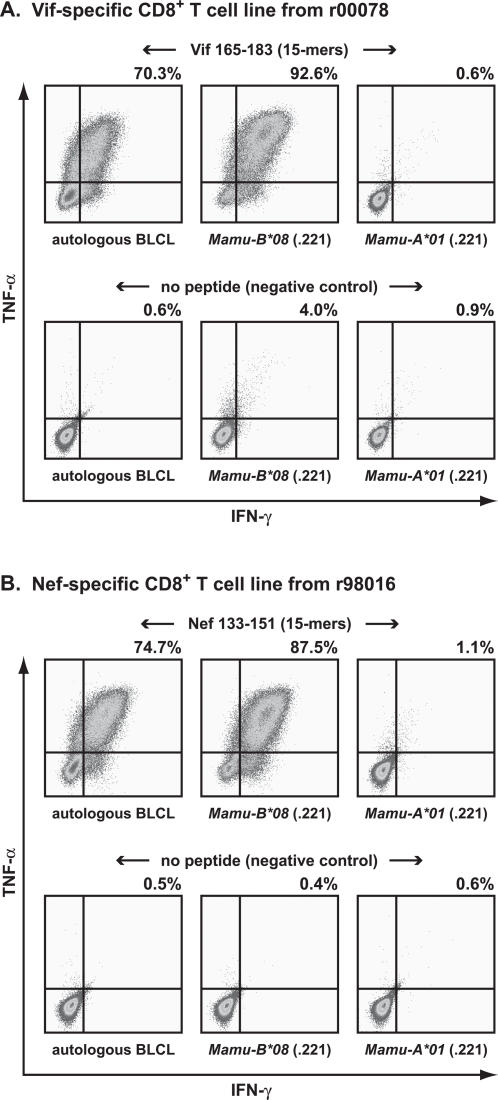
Mamu-B*08 restricts both of the novel SIV-specific CD8^+^ T cell responses observed in the CD8^+^ cell-depleted EC macaques. 721.221 cells, which do not express endogenous classical MHC class I molecules, were stably transfected with Mamu-B*08 or Mamu-A*01 and pulsed with the appropriate peptides. Peptide-pulsed cells were used in ICS assays with peptide-specific CD8^+^ T cell lines. CD8^+^ T cell lines raised against 15-mers derived from A) Vif (positions 165–183) from animal r00078 and B) Nef (positions 133–151) from animal r98016 were stimulated by the Mamu-B*08 stable transfectants pulsed with the relevant peptides. The cytokine reactivity was comparable to levels elicited when autologous BLCL were used as APCs (positive control). No peptide stimulation was observed when using the Mamu-A*01 stable transfectants as APCs (negative control). Negative controls without peptide in the presence of APCs also gave negligible cell stimulation. Percentages over each FACS dot plot signify the maximal cytokine response (total of IFN-γ^+^ cells, TNF-α^+^ cells, and IFN-γ^+^ TNF-α^+^ cells).

To test the hypothesis that Mamu-B*08 presented the novel epitopes, we next generated CD8^+^ T cell lines from the three ECs using 15-mer peptides containing the Vif or Nef epitopes. We also stably transfected 721.221 cells [66], [67] with expression constructs for Mamu-B*08 or Mamu-A*01. 721.221 is a human cell line that does not express classical MHC class I molecules, so the only MHC class I molecules on the surface of the transfectants were the products of the transfected constructs. We then asked whether Mamu-B*08 transfectants could present exogenous peptides and elicit cytokine responses detectable in ICS assays. Indeed, CD8^+^ T cell lines responded at least as strongly to Mamu-B*08-transfectant cells as they did to autologous B-lymphoblastoid cell lines (BLCL) pulsed with either the Vif peptides (Fig. 3A) or the Nef peptides (Fig. 3B). The CD8^+^ T cell lines did not respond to mismatched Mamu-A*01 transfectants pulsed with the same peptides (Fig. 3A and 3B) or to peptide-pulsed 721.221 cells (data not shown). Taken together, these data indicate that Mamu-B*08 presents the novel epitopes derived from SIV Vif and Nef.

### Identification of five more Mamu-B*08-restricted CD8^+^ T cell epitopes derived from SIV_mac_239

The Nef epitope we identified, Nef_137–146_RL10 (RRHRILDIYL), overlapped with a previously identified epitope, Nef_136–146_AL11 (ARRHRILDIYL), which is restricted by Mamu-B*03 [22], [67]. Therefore, we compared the amino acid sequences of these two MHC class I molecules to determine whether we could predict a peptide binding motif for Mamu-B*08 extrapolated from the known motif of Mamu-B*03 [75]. Mamu-B*03 and Mamu-B*08 are almost identical in amino acid sequence [74]. There are only two amino acid differences between these molecules in regions that influence peptide binding and antigen recognition [76], [77]. Both differences reside in the alpha-1 domain (exon 2). Thus, based on these overall structural similarities, analysis of the B and F pockets of Mamu-B*08 in comparison with well-characterized HLA and Mamu specificities, and the sequences of known Mamu-B*03 [22], [67] and the identified Mamu-B*08 epitopes (Figs. 1 and 2), a preliminary Mamu-B*08 peptide binding motif was derived. This preliminary motif specifies the presence of arginine (R) in position 2 (P2) and the aliphatic hydrophobic residues leucine (L), isoleucine (I), or valine (V) at the C-terminus.

**Figure 4:**
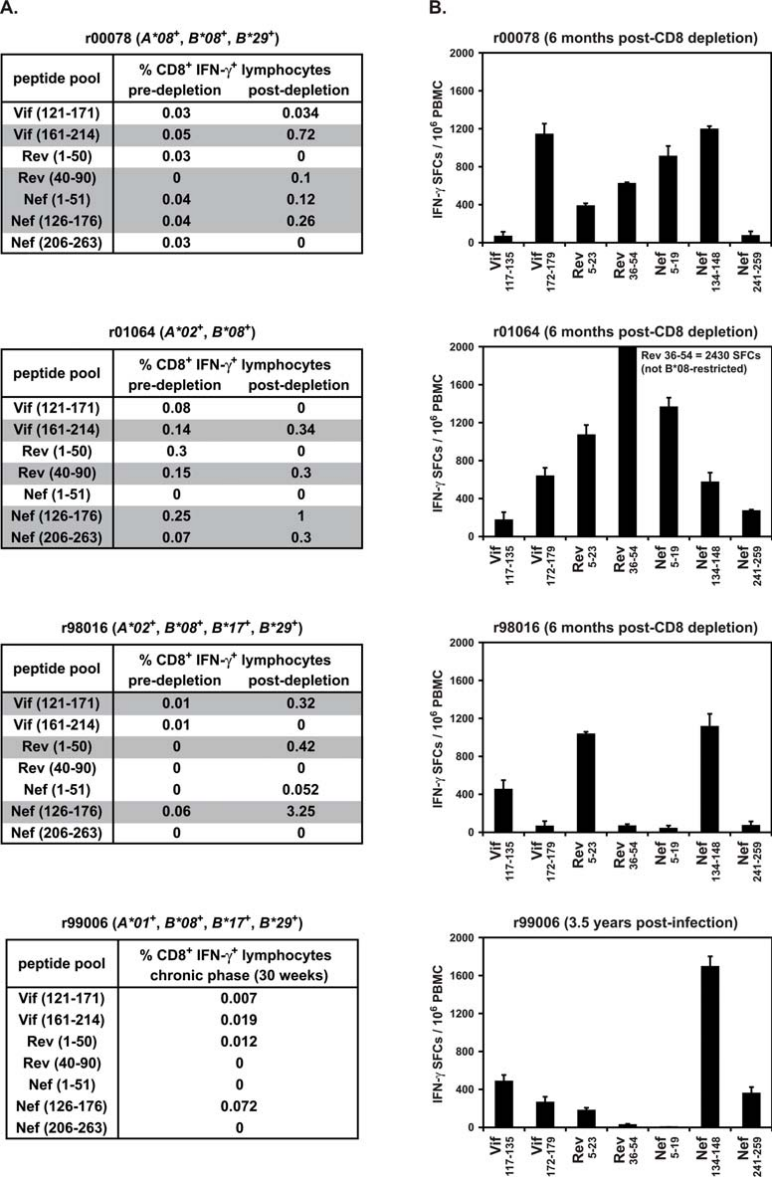
Expansion and persistence of seven SIV-specific Mamu-B*08-restricted CD8^+^ lymphocyte responses in four EC macaques. MHC class I alleles detected by PCR-SSP are listed for each animal. A) Unknown CD8^+^ responses from *ex vivo* ICS assays using SIV peptide pools (containing ten 15-mers overlapping by eleven amino acids) performed one month prior to and four weeks after CD8^+^ cell depletion. Chronic phase (thirty weeks post-infection) *ex vivo* ICS data is also shown for responses in r99006 to the seven SIV 15-mer peptide pools that contain the novel CD8^+^ T cell epitopes. Grayed responses increased >0.1% after CD8^+^ cell depletion (r00078, r01064, and r98016). B) *Ex vivo* IFN-γ ELISPOT using one or two overlapping 15-mers containing the minimal optimal Mamu-B*08-restricted SIV-specific epitopes (or the minimal optimal peptide alone, in the case of Vif_172–179_RL8) demonstrated that these CD8^+^ responses persist approximately six months post-CD8^+^ cell depletion in ECs r00078, r01064, and r98016. CD8^+^ cell responses persist in r99006 at ∼3.5 years post-SIV infection. Mean values and SD from triplicate wells were calculated for each ELISPOT assay. Background (the mean of wells without peptide) levels were subtracted from each well. Mean responses <50 SFC per 1×10^6^ cells were not considered positive because these counts were not significantly above background.

We then used the preliminary binding motif to scan SIV_mac_239 Vif, Nef, and Rev for additional potential Mamu-B*08-restricted epitopes. We focused in particular on these regions because CD8^+^ T cell populations recognizing these three proteins expanded in r00078, r01064, and r98016 after CD8^+^ cell depletion (Fig. 4A). We used ELISPOT assays to test the ability of 15-mer peptides containing sequences that fit the motif to stimulate IFN-γ release by PBMC from these three ECs.

We also determined that an additional animal in the EC cohort, r99006, expressed Mamu-B*08, so this animal was included in further analyses. r99006 was challenged with a recombinant virus bearing escape mutations in Mamu-A*01- and Mamu-B*17-restricted CD8^+^ T cell epitopes as part of a prior study [59], [60]. This animal has controlled viremia to <1,000 vRNA copy Eq/ml for over four years post-SIV infection and was not part of the CD8^+^ cell depletion study. Responses to four peptide pools containing putative Mamu-B*08-restricted epitopes were weakly detectable in ICS assays of r99006 PBMC conducted 30 weeks post-infection (Fig. 4A).

Testing PBMC from all four ECs, we identified another five putative Mamu-B*08-restricted CD8^+^ T cell epitopes derived from Vif, Nef, and Rev (Fig. 4B). TNF-α and IFN-γ ICS assays using Mamu-B*08 and Mamu-A*01 transfectants confirmed that each of these epitopes was presented by Mamu-B*08 (data not shown). For each Mamu-B*08-restricted response detected by 15-mers, we selected candidate peptides within the 15-mers to define the minimal optimal epitopes. These candidate peptides were chosen on the basis of the preliminary motif and tested in serial dilutions in TNF-α and IFN-γ ICS assays with peptide-specific CD8^+^ T cell lines as described above for Vif_172–179_RL8 and Nef_137–146_RL10 (data not shown). All seven minimal optimal epitopes had an R residue at P2 and L at the C-terminus, in agreement with the putative Mamu-B*08 binding motif (Table 2).

**Table 2:** Summary of seven novel SIV-derived epitopes restricted by Mamu-B*08 that were identified in four EC macaques.

Protein	Amino Acid Positions	Length	Short Name	Sequence
Vif	123–131	9	RL9	RRAIRGEQL
Vif	172–179	8	RL8	RRDNRRGL
Rev	12–20	9	KL9	KRLRLIHLL
Rev	44–51	8	RL8	RRRWQQLL
Nef	8–16	9	RL9	RRSRPSGDL
Nef	137–146	10	RL10	RRHRILDIYL
Nef	246–254	9	RL9	RRLTARGLL

Surprisingly, a majority of the novel Mamu-B*08-restricted CD8^+^ T cell responses were detected in at least two ECs over 1.5 years after initial infection with SIVmac239 (Fig. 4B). Among these responses, populations recognizing the Nef_137–146_RL10 epitope appeared dominant or co-dominant in three of four ECs tested. The exception to this pattern was animal r01064, whose Mamu-B*08-restricted response appeared to be dominated by cells responding to an epitope in the C-terminus of Rev, Rev_44–51_RL8. However, the overlapping Rev 15-mers tested contain not only a Mamu-B*08-restricted CD8^+^ T cell epitope but also a CD8^+^ T cell epitope of unknown restriction, which may contribute to the strength of immune responses elicited by this peptide (data not shown). Further experiments suggested that the strong response to the Rev 15-mer we observed in r01064 is due to cells that recognize this unknown epitope (see below).

### Staining PBMC with Mamu-B*08 tetrameric complexes reveals the immunodominance hierarchy of Mamu-B*08-restricted CD8^+^ T cell responses

Having defined seven minimal optimal epitopes bound by Mamu-B*08, we next produced tetrameric complexes of Mamu-B*08 loaded with each of these epitopes. The PCR-SSP-based screen for Mamu-B*08 we developed allowed us to test for the presence of this allele in PBMC from 192 SIV_mac_239-infected rhesus macaques [54]. Seven animals from this cohort expressed Mamu-B*08: four of these were the previously identified ECs, and three were animals that progressed to AIDS [17], [54].

We used the set of Mamu-B*08 tetramers to measure the Mamu-B*08-restricted CD8^+^ T cell response in archived chronic-phase PBMC from six of these seven SIV-infected macaques. For each epitope, there were detectable tetramer-binding populations of CD8^+^ T cells in at least two animals (Table 3). In most instances, the frequency of SIV-specific CD8^+^ T cells against each of the seven novel Mamu-B*08-restricted epitopes was higher in the four EC macaques than the two progressors. Populations recognizing Nef_137–146_RL10 were dominant in one of the six macaques (r99006), and co-dominant in three more animals with Vif_172–179_RL8 (r96104) and with Rev_12–20_KL9 (r98016, r96113). Nef_8–16_RL9-specific cells were dominant or co-dominant in the remaining two animals (r00078 and r01064). The Nef_245–254_RL9 was co-dominant with the Nef_8–16_RL9-specific in r00078.

**Table 3:** Comparison of CD8^+^ T cell responses detected by MHC class I tetramers folded with the seven Mamu-B*08-restricted SIV-specific epitopes reveals the chronic phase immunodominance hierarchy.

Animal #	Timepoint	% CD3^+^ CD8^+^ tetramer^+^ gated lymphocytes^a^						
		Vif (123–131) RL9	Vif (172–179) RL8	Rev (12–20) KL9	Rev (44–51) RL8	Nef (8–16) RL9	Nef (137–146) RL10	Nef (246–254) RL9
r00078	∼6 months post-CD8^+^ cell depletion	0.064	0.9	0.23	0.54	1.13	0.87	1.16
r01064	∼6 months post-CD8^+^ cell depletion	0.24	1.07	1.58	-	2.5	0.39	0.87
r98016	∼6 months post-CD8^+^ cell depletion	0.46	0.05	1.34	-	0.041	1.06	0.059
r99006	∼3.5 years post-SIV infection	0.38	0.26	0.18	-	-	1.8	0.13
r96104	TOD (60 wks post-SIV infection)	-	0.1	-	-	0.04	0.088	0.033
r96113	TOD (75 wks post-SIV infection)	0.034	0.055	0.21	0.069	-	0.2	0.023
	**# of responders**	**5**	**6**	**5**	**2**	**4**	**6**	**6**

a) Tests in which the frequency of tetramer-binding cells was <0.02% are displayed as a dash (–).

The high frequency populations detected by Mamu-B*08 Nef_137-146_RL10 tetramers also confirmed that responses we detected by IFN-γ ELISPOT in four Mamu-B*08-positive ECs were directed against this Mamu-B*08-restricted epitope (Fig. 4B). Indeed, there was general concordance between the immunodominance hierarchy detected by ELISPOT and that measured by tetramers with one exception. In animal r01064 using the Rev_44–53_RL8 tetramer, we did not detect a CD8^+^ T cell response (Table 3), although 15-mer peptides containing this sequence elicited the dominant response in ELISPOT assays (Fig. 4B). This result further suggests that the strong response detected in ELISPOT is due to a population that recognizes an epitope not presented by Mamu-B*08.

### Mamu-B*08-restricted CD8^+^ T cells select for viral variation in several SIV_mac_239 epitopes

**Figure 5:**
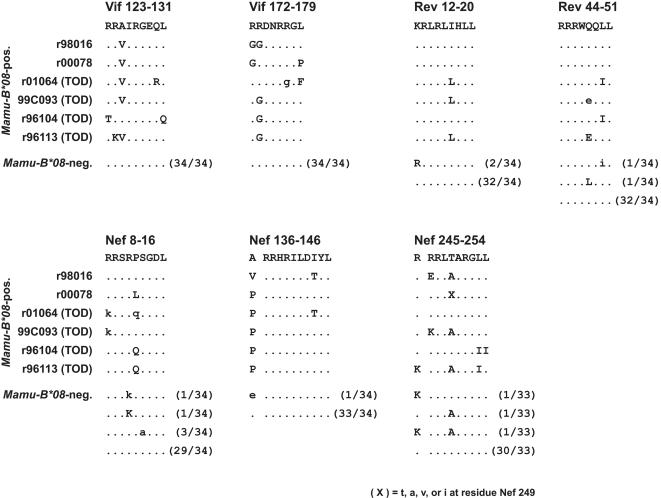
Amino acid variation in novel epitope sequences is associated with expression of Mamu-B*08. Viral RNA was isolated from cell-free plasma and directly sequenced. Samples were taken from the time of necropsy for four Mamu-B*08-positive macaques that were sacrificed with progressive SIV_mac_239 infection (time of necropsy indicated for each animal): r01064 (77 weeks post-CD8^+^ cell depletion, 129 weeks post-SIV infection), 99C093 (127 weeks post-SIV infection), r96104 (60 weeks post-SIV infection), and r96113 (75 weeks post-SIV infection). Virus was sampled from Mamu-B*08-positive EC r00078 78 weeks post-CD8^+^ cell depletion (161 weeks post-infection) after viral breakthrough. Virus was sampled from Mamu-B*08-positive EC r98016 during a transient increase in SIV replication at 48 weeks post-CD8^+^ cell depletion (164 weeks post-infection) during which virus load rose to ∼9,000 vRNA copy Eq/ml before returning to <1,000 copy Eq/ml. Viral sequences from Mamu-B*08-positive animals were compared to sequences obtained at the time of necropsy from 34 Mamu-B*08-negative progressors [68]. The frequency of each sequence detected in these 34 animals is listed in parentheses except for Nef 245–254 where sequences were available from 33 of the 34 animals. Amino acid residues immediately N-terminal to two Nef epitopes are listed and denoted with a space. Those amino acids identical to the wild-type sequence are shown as dots. Complete amino acid replacements are shown in uppercase; sites of mixed-base heterogeneity are shown in lowercase.

To determine whether viral variation in CD8^+^ T cell epitopes could be associated with expression of Mamu-B*08, we first sequenced *vif*, *rev*, and *nef* ORFs in virus isolated from plasma of chronically infected Mamu-B*08-positive controllers and progressors. We compared the predicted amino acid sequences of the epitope regions and full-length proteins to sequences previously obtained at the time of necropsy from 34 Mamu-B*08-negative MHC-defined macaques infected with SIV_mac_239 [68]. Strikingly, amino acid substitutions accumulated in each of the seven Mamu-B*08-restricted epitopes we identified in at least two Mamu-B*08-positive macaques (Fig. 5). The substitutions were rarely in residues likely to be crucial for binding to the Mamu-B*08 molecule, P2 or the C-terminus. One exception to this pattern was Vif_172–179_RL8, in which substitutions affected either P2 or the C-terminus in each animal with epitope mutations. Peptides with P2 glycine (G)-for-arginine (R) substitutions were not recognized by Vif_172–179_RL8-specific CD8^+^ T cell lines in ICS assays, supporting the conclusion that these mutations confer escape from Mamu-B*08-restricted CD8^+^ T cell responses (data not shown).

In order to examine the effects of selection pressure on Mamu-B*08-restricted epitope sequences more closely, we compared the numbers of nonsynonymous (*d_N_*) and synonymous (*d_S_*) substitutions per site within epitope-coding regions of *vif*, *rev*, and *nef* with *d_N_* and *d_S_* for the entire ORFs. This analysis showed that *d_N_* was significantly elevated in regions encoding Mamu-B*08-restricted epitopes in animals expressing this molecule (*P*<0.001, Table 4). Furthermore, *d_N_* within the epitope regions was greater in Mamu-B*08-positive than in Mamu-B*08-negative animals (*P*<0.001), while *d_S_* was not significantly different between the groups (Table 4). These results suggest that regions encoding Mamu-B*08-restricted CD8^+^ T cell epitopes are under selective pressure only in the presence of Mamu-B*08, and thus the variation we detect in Mamu-B*08-restricted epitopes is indeed selected by CD8^+^ T cell responses.

**Table 4:** Comparison of *d_N_* and *d_S_* for the novel Mamu-B*08-restricted CD8^+^ T cell epitopes in Mamu-B*08-positive and Mamu-B*08-negative SIVmac239-infected macaques.

Mamu-B*08 expression	Region of viral ORFs sequenced^a^	Mean *d_N_*±S.E.	Mean *d_S_*±S.E	*d_N_ = d_S_?* b
**positive (6 macaques)**	B*08-restricted epitopes^c^	0.0556±0.0011	0.0164±0.0075	*P*<0.001
	Remainder	0.0072±0.0011	0.0041±0.0015	n. s.
**negative (34 macaques)**	B*08-restricted epitopes^c^	0.0028±0.0008	0.0014±0.0007	n. s.
	Remainder	0.0063±0.0006	0.0025±0.0006	*P*<0.001
B*08 **+ ** ***d_N_*** ** = ** B*08 **- ** ***d_N_*** **?** d	B*08-restricted epitopes^c^	*P*<0.001		
B*08 **+ ** ***d_S_*** ** = ** B*08 **- ** ***d_S_*** **?** d	B*08-restricted epitopes^c^		n.s.	

**Figure 6:**
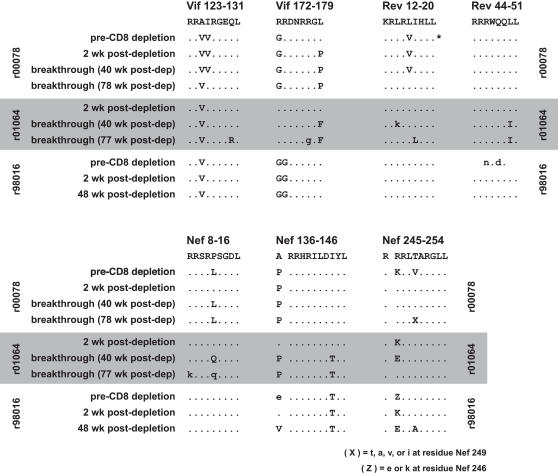
Amino acid variation accumulates in the seven Mamu-B*08-restricted CD8^+^ T cell epitopes before and after CD8^+^ cell depletion in three Mamu-B*08-positive EC macaques. Virus was isolated from r00078, r01064, and r98016 at the indicated times before and after CD8^+^ cell depletion. Pre-depletion samples were obtained one month prior to depleting antibody treatment, when virus loads were <500 vRNA copy Eq/ml. An asterisk (*) indicates a Rev sequence obtained five months prior to CD8^+^ cell depletion in animal r00078. Pre-depletion sequence from r98016 containing Rev_44–51_RL8 could not be amplified despite repeated attempts and is listed as n.d. (not done). Viral RNA from pre-depletion plasma samples from r01064 could also not be amplified despite repeated attempts due to low concentrations of vRNA. Viral RNA was isolated from cell-free plasma and analyzed by direct sequencing of viral amplicons spanning the SIV genes *vif*, *rev*, and *nef*. Amino acids residues immediately N-terminal to two Nef epitopes are listed and denoted with a space. Those amino acids identical to the wild-type sequence are shown as dots. Complete amino acid replacements are shown in uppercase; sites of mixed-base heterogeneity are shown as lowercase.

a) We sequenced the entire open reading frames (ORFs) of *vif*, *rev*, and *nef* from chronic infection or at time of necropsy in 6 Mamu-B*08-positive and 34 Mamu-B*08-negative macaques infected with SIVmac239. We compared epitope-encoding regions to the remainder of the three ORFs using the following tests.

b) Paired t-test (2-tailed) performed to determine whether *d_N_* was equal to *d_S_* in Mamu-B*08-restricted CD8^+^ T cell epitope sequences and in non-Mamu-B*08-restricted epitope regions. (n.s. = not significant)

c) We included one amino acid N-terminal to the Nef epitopes RL10 (pos. 137–146) and RL9 (pos. 246–254) because it appeared that variation was associated with Mamu-B*08 expression. See text and Figs. 5 and 6 for details.

d) Two-sample t-tests (2-tailed) were performed to determine whether *d_N_* and *d_S_* values were significantly higher in Mamu-B*08-positive animals than in Mamu-B*08-negative animals. (n.s. = not significant)

In one case, substitutions associated with Mamu-B*08 expression occurred not in the epitope sequence, but immediately N-terminal to it. Virus from each of the Mamu-B*08-positive macaques tested had a substitution at Nef residue 136, which is alanine (A) in SIV_mac_239. The variant sequence encoded a proline (P) at this position (A136P) in five of six animals (Fig. 5). The *d_N_*/*d_S_* ratio was significantly elevated at this position only in Mamu-B*08-positive animals, indicating strong selection pressure favoring this mutation only in the presence of Mamu-B*08 (data not shown). Since Nef residue 136 is not within the minimal optimal epitope bound by Mamu-B*08, it is possible that A136P affects peptide processing, decreasing the availability of minimal optimal epitopes to Mamu-B*08 molecules. Indeed, a similar A to P substitution immediately N-terminal to the epitope is known to inhibit processing of an HLA-B57-restricted peptide in HIV Gag [78]. At position 136, the A to valine (V) substitution found in EC r98016 is much more conservative and may not alter processing of the epitope. We also detected a substitution one amino acid N-terminal of the minimal optimal Nef_246–254_RL9 epitope in progressor r96113 (Fig. 5). This was a conservative lysine (K)-for-R substitution, so the impact of this change on peptide processing may be small. This same substitution was also found in two of 33 Mamu-B*08-negative macaques, and relative rates of nonsynonymous and synonymous substitutions were not significantly different in this epitope region in Mamu-B*08-positive and Mamu-B*08-negative animals (data not shown). These data suggest that the R245K substitution is not selected for by Mamu-B*08-restricted CD8^+^ T cells.

To clarify whether substitutions in Mamu-B*08-restricted epitopes might affect the ability of Mamu-B*08-positive animals to control SIV_mac_239 infection, we next determined epitope sequences of viruses circulating in Mamu-B*08-positive ECs before and after CD8^+^ cell depletion, when SIV replication was effectively controlled (Fig. 6). We compared these sequences to those present in virus replicating in the absence of CD8^+^ T cell selection during the CD8^+^ cell depletion phase, which we had determined previously [55]. To our surprise, variation in several Mamu-B*08-restricted epitopes was detectable in ECs r98016 and r00078 even before CD8^+^ cell depletion, when viremia had been successfully controlled to <1,000 vRNA copy Eq/ml for up to two years (Fig. 6). Repeated attempts to amplify viral cDNA from r01064 pre-depletion plasma samples were unsuccessful, likely due to the extremely low concentrations of vRNA in this animal.

Viral variation was detectable prior to depletion in five of seven identified Mamu-B*08-restricted epitopes in r00078, and in four of six epitopes for which sequence was available in r98016. Mutations present before depletion were still detectable during the peak of virus replication, with three exceptions. Mutations detected in animal r00078 Nef_246–254_RL9 were not present at peak, although at least one of these mutations, encoding R246K, was detected in the two other ECs' peak virus (Fig. 6). Also in animal r00078, a leucine (L)-for-P substitution was also detected at position five of Nef_8–16_RL9 before CD8^+^ cell depletion, but not in the virus replicating in the absence of CD8^+^ cells. This same substitution reappeared in virus populations isolated at later timepoints, after the return of CD8^+^ cells. Virus from animal r01064 harbored mutations in the same codon, resulting in a different amino acid substitution (P to glutamine, Q). This change was similarly absent from virus replication during CD8^+^ cell depletion. In addition, a pre-depletion site of mixed-base heterogeneity N-terminal to Nef_137–146_RL10 in r98016, encoding A and glutamic acid (E), was fully wild type at the peak of virus replication. In contrast, r00078's virus at position 136 had an A-to-P mutation before, during and after CD8^+^ cell depletion. These results suggest that commonly detected mutations within these Mamu-B*08-restricted epitopes do not likely exact a heavy cost to viral fitness.

The two Mamu-B*08-positive, Mamu-B*17-negative ECs eventually lost control of viremia, with plasma virus concentrations increasing beyond 1,000 vRNA copy Eq/ml beginning ∼150 days after CD8^+^ cell depletion treatment. Animal r00078's viremia stabilized at a new plateau level of ∼10,000 vRNA copy Eq/ml by day 300 after depletion, but r01064 had steadily increasing viremia and was sacrificed at day 542 post-depletion with a plasma virus concentration of 1×10^6^ vRNA copy Eq/ml (data not shown). We sequenced plasma virus at two timepoints following this virus breakthrough in both animals to determine whether variation in Mamu-B*08 epitopes could account for their loss of control. Although the patterns of variation were different in each animal, post-breakthrough virus had substitutions that had not been detected immediately after CD8^+^ cell depletion (Fig. 6). Although we cannot yet determine which substitutions in particular Mamu-B*08-restricted CD8^+^ T cell epitopes can be correlated with loss of effective viremia control, we were surprised to find that Mamu-B*08-restricted CD8^+^ T cell responses appeared to select for viral variation in all identified epitopes in the majority of animals.

## Discussion

In a recent study aimed at identifying correlates of EC of SIV replication, we transiently depleted CD8^+^ cells from the periphery in six macaque ECs [55]. Four of these animals expressed Mamu-B*17, which we had previously shown to be associated with a reduction in chronic phase viremia in a large cohort of SIV_mac_239-infected macaques [17]. In that study, we found that particular epitope-specific populations of CD8^+^ T cells expanded dramatically as CD8^+^ cells repopulated the periphery and control of virus replication was re-established. In the Mamu-B*17-positive animals these expanding populations frequently targeted previously described Mamu-B*17-restricted epitopes in Vif and Nef. To our surprise, the Mamu-B*17-negative animals in that study also had expanding populations of CD8^+^ T cells that targeted Vif and Nef (Figs. 1 and 2). Because these populations showed a substantial expansion as control of SIV replication was re-asserted, we speculate that they played an important role in this control.

Here we show that these expanding CD8^+^ T cell populations in the Mamu-B*17-negative macaques recognized novel epitopes bound by the MHC class I molecule Mamu-B*08 (Fig. 3). Indeed, Mamu-B*08 presents at least seven epitopes derived from SIV_mac_239 to CD8^+^ T cells (Table 2) and is expressed in three of the six ECs in our CD8^+^ cell depletion study, including both Mamu-B*17-negative ECs. We recently reported that Mamu-B*08 was enriched in a cohort of EC macaques and is associated with reduced chronic phase viremia in SIV_mac_239-infected rhesus macaques [54], further indicating that Mamu-B*08-restricted CD8^+^ T cell responses play an important role in controlling SIV replication. Using cytokine secretion assays and synthesizing Mamu-B*08 tetramers, we found strong responses to the newly defined epitopes in chronically infected ECs, even when viremia was low. Among the newly identified epitopes were two derived from SIV_mac_239 Rev, Rev_12-20_KL9 and Rev_44-51_RL8. These are the first MHC class I minimal optimal epitopes described in SIV Rev.

Cytokine secretion assays and MHC class I tetramer stains also allowed us to identify immunodominance hierarchies among Mamu-B*08-restricted CD8^+^ T cell populations (Fig. 4 and Table 3). Nef_137–146_RL10-specific CD8^+^ T cells were dominant or co-dominant as detected by MHC class I tetramers in four of six Mamu-B*08-positive animals in the chronic phase of SIV infection. Strong IFN-γ responses to Nef_137–146_RL10-containing peptides were also detected in all four Mamu-B*08-positive ECs. CD8^+^ T cell populations responding to Rev or Vif epitopes were co-dominant with or subdominant to these Nef_137-146_RL10-specific populations in the three ECs.

Sequencing of *vif*, *rev*, and *nef* ORFs revealed that Mamu-B*08-restricted CD8^+^ T cells exert selective pressure on a surprisingly broad scale. All seven epitopes we identified had substitutions in at least two of seven chronically SIV-infected Mamu-B*08-positive animals (Fig. 5). Substitutions within the dominant Nef_137–146_RL10 epitope sequence were infrequent in these animals, but a mutation one codon upstream of the epitope (encoding A136P) occurred in six of seven Mamu-B*08-positive animals. A study of HIV-infected patients showed that viruses bearing a similar alanine-to-proline substitution immediately N-terminal of an HLA-B57-restricted Gag epitope were not recognized by epitope-specific CTL [78]. In that case, the A-to-P mutation altered processing of the epitope peptide, decreasing its availability for loading onto HLA-B57. It is possible that the A136P substitutions we observed in Nef similarly alter processing of the Mamu-B*08-restricted epitope peptide. We detected evidence for positive selection pressure on Mamu-B*08-restricted CD8^+^ T cell epitopes associated with expression of Mamu-B*08 (Table 4). Therefore, it is likely that these substitutions represent viral escape from Mamu-B*08-restricted CD8^+^ T cell responses. Viral escape in particular CD8^+^ T cell epitopes has previously been correlated with a loss of effective immune containment of viremia in *HLA-B27*-positive humans and Mamu-A*01-positive macaques [21], [24], [49]–[51]. In our study, it was difficult to correlate particular epitope substitutions with the eventual loss of control of viral replication in two CD8^+^ cell-depleted Mamu-B*08-positive ECs (Fig. 6).

It is thought that individuals with “elite” control of HIV infection provide an important and all-too-rare example of successful host responses to the virus. Although these individuals have been studied for at least ten years, the mechanisms of control have been extremely difficult to define. Expression of particular MHC class I alleles, particularly *HLA-B27* and -*B57*, has been associated with elite control of HIV infection [7]–[12]. It seems reasonable to infer from this association that certain HLA-B27- and HLA-B57-restricted epitope-specific CD8^+^ T cells are particularly effective at controlling virus replication. But so far, with the possible exception of the HLA-B27-restricted epitope Gag_263-272_KK10, no candidate “controller” responses have been identified. MHC class I alleles may exert their protective effects through additional, as yet unknown, means.

Animal models of MHC class I-associated elite control of immunodeficiency virus replication could therefore offer a valuable tool for studying the mechanisms of control. We have previously shown that Mamu-B*17 is over-represented in a cohort of SIV_mac_239 ECs [17]. Recently, we discovered that Mamu-B*08 expression is associated with a reduction in chronic phase viremia of a similar magnitude to that seen for Mamu-B*17 [54]. Interestingly, the peptide binding motif of Mamu-B*17 is broadly similar to that of HLA-B57. Although Mamu-B*17 seems to tolerate a wider array of residues at P2, both molecules exhibit a preference for W, F, or Y at the C-terminus [73], [79], [80]. At the same time, a preliminary binding motif for Mamu-B*08, defined on the basis of structural analyses, is similar to that defined for HLA-B27 [74], [75], [79], [80]. All seven Mamu-B*08-restricted CD8^+^ T cell epitopes we identified contained R at position 2 and L at the C-terminus, which also fits the HLA-B27 motif. Indeed, a recent study has provided evidence that this P2 requirement for R, a long, basic residue, may result in some unusual peptide binding and presentation characteristics for HLA-B27. Peptides with a dibasic N-terminal motif were relatively resistant to degradation by cytosolic aminopeptidases, resulting in an over-representation of peptides with the N-terminal motif KR or RR in the HLA-B27 peptidome [81]. The authors of that study suggested that because HLA-B27 “selects” ligands that are particularly stable in the cytosol. It therefore may require fewer molecules of viral peptides than other HLA class I molecules to trigger a response from CD8^+^ T cells. This might help explain the apparent effectiveness of HLA-B27-restricted CD8^+^ T cell responses against HIV. Since Mamu-B*08 shares the peptide-binding characteristics of HLA-B27, it may similarly restrict CD8^+^ T cells capable of recognizing infected cells even when cytosolic virus-derived peptide concentrations are low, for example immediately after infection, or in latent infection.

As the HIV pandemic progresses, so does the urgent need for an AIDS vaccine. Although there is broad agreement that CD8^+^ T cell responses will be an important component of vaccine-induced immunity, the attributes of effective antiviral CD8^+^ T cells are still unknown. Since they appear naturally to make effective immune responses, ECs, both macaque and human, provide an important example of what is possible. Here we report a new model of elite control of immunodeficiency virus infection, Mamu-B*08-positive macaques that effectively control infection with the pathogenic clone SIV_mac_239. It will be important in future studies to refine the Mamu-B*08 peptide binding motif, to identify all of the SIV-derived epitopes bound by this molecule, and to test the efficacy of Mamu-B*08-restricted CD8^+^ T cells more directly. Interestingly, Mamu-B*08-associated control of SIV_mac_239 replication may closely parallel *HLA-B27*-associated control of HIV replication.
